# Ubiquitin System-Driven Proteostasis in DNA Damage Response

**DOI:** 10.3390/ijms27052356

**Published:** 2026-03-03

**Authors:** Renata Kusuma, Audrey Regina Valerie, Sisi Qin, Kitty Ichiwa, Kenneth Joshua, Gayoung Seo, Wootae Kim

**Affiliations:** 1 Department of Integrated Biomedical Science, Soonchunhyang Institute of Medi-bio-Science (SIMS), Soonchunhyang University, Cheonan 31151, Republic of Korea20259199@sch.ac.kr (A.R.V.);; 2 Institute for Molecular Metabolism Innovation, Soonchunhyang University, Asan 31538, Republic of Korea; 3 Department of Pathology, University of Chicago, Chicago, IL 60637, USA; sisiq@uchicago.edu

**Keywords:** proteostasis, ubiquitin-system, DNA damage response, genomic instability, cancer therapy

## Abstract

Proteostasis is essential for maintaining the proper function of the proteome and diverse cellular processes. The ubiquitin system plays a central role in proteostasis by regulating protein stability, trafficking, and termination. Under cellular stress, rapid proteome remodeling is required to maintain proteostasis and support adaptive cellular stress-response pathways, including the DNA damage response (DDR). Proper DDR function relies on precise control of protein abundance and signaling dynamics, primarily achieved through ubiquitin-mediated proteostatic regulation involving both proteolytic degradation and non-proteolytic scaffolding function. Dysregulation of the ubiquitin system alters the dynamic control of the DDR cascade, leading to genomic instability and disease progression. Therefore, targeting key components of the ubiquitin system may restore proper DDR signaling regulation and offer novel therapeutic opportunities for disease treatment. In this review, we summarize the role of the ubiquitin system in proteostasis-mediated DDR regulation and explore the potential of targeting ubiquitin system components as therapeutic strategies in cancer treatment.

## 1. Introduction

**Figure 1:**
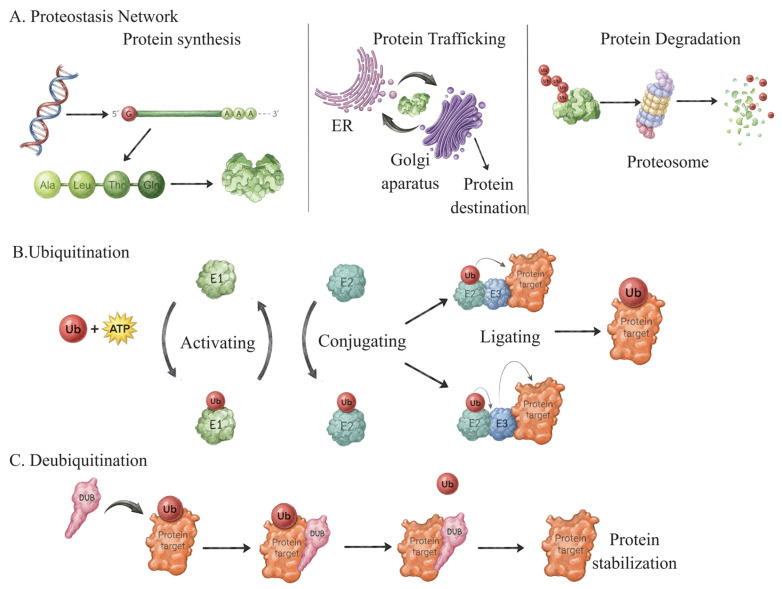
**Overview of the proteostasis network and ubiquitin system.** (**A**) The proteostasis network maintains protein homeostasis by coordinating protein synthesis, intracellular trafficking, and proteasomal degradation. (**B**) The ubiquitin system regulates protein homeostasis and fate through the E1-E2-E3 enzymatic cascade, resulting in mono- or polyubiquitination, which controls protein stability, localization, and activity. (**C**) DUBs reverse ubiquitination, ensuring dynamic control of protein turnover and proteostasis.

Proteins are fundamental building blocks of life that carry out diverse biological processes within cells, including signaling, metabolism, and stress responses. Given their central role, cells employ coordinated regulatory pathways to modulate protein availability and activity—a process known as proteostasis [1,2]. The proteostasis network (PN) constitutes a multi-compartment, highly interconnected system that orchestrates protein stabilization, trafficking, and termination (Figure 1) [1,2,3]. Through precise regulation of protein availability and functional persistence, the PN ensures that proteins are stabilized and remain active in a context-dependent manner [4]. At the same time, proteins that are damaged or no longer required are selectively targeted for removal or degradation [5,6,7]. Additionally, the PN organizes protein distribution and recruitment to specific cellular compartments [5,6,7]. Collectively, these processes maintain both proteome integrity and cellular homeostasis [1].

The ubiquitin system serves as a core executor of proteostasis. It involves two major processes, ubiquitination and deubiquitination, which act dynamically to direct proteins toward either stabilization or degradation, depending on the cellular (Figure 1) [8]. Ubiquitination occurs through a three-step enzymatic cascade mediated by ubiquitin-activating enzyme (E1), ubiquitin-conjugating enzyme (E2), and ubiquitin ligase (E3), which work coordinately to attach ubiquitin to substrate proteins [8,9,10]. On the other hand, the deubiquitinase (DUB) protein family removes ubiquitin modifications from the substrates [8,10]. The interplay between ubiquitin ligases and deubiquitinases provides reversible control over protein fate, allowing cells to fine-tune proteostasis in response to cellular demand and stress. The equilibrium state of both key processes ensures precise control of protein turnover, safeguarding appropriate protein abundance and function [8,9,10,11].

Cells are constantly exposed to stressors that can lead to the formation of DNA damage. To maintain genomic integrity, they have evolved a coordinated surveillance and repair system known as DNA damage response (DDR) [12,13]. DDR is a highly dynamic signaling network that requires rapid activation and timely resolution to preserve genome integrity. Dysregulation of DDR is closely associated with various diseases, including cancer, neurological disorders, and aging. Therefore, cells tightly regulate DDR to ensure it is activated only when DNA damage occurs and deactivated once the repair process is complete [12,13,14].

Emerging evidence has demonstrated that proteostasis is closely linked to DDR signaling [15]. Disruption of proteostasis hinders DNA damage repair capacity, leading to the accumulation of DNA lesions and genomic instability [15,16]. Proteostasis provides a regulatory framework that can either stabilize DDR proteins or actively terminate them via protein degradation, thereby determining the functional lifespan of the DDR machinery [2,3,17]. In particular, the ubiquitin system safeguards DDR pathways through both proteolytic and non-proteolytic mechanisms that constantly modulate not only protein degradation but also spatial recruitment and signaling dynamics of DDR proteins [4].

This review summarizes emerging insights into ubiquitin-mediated control of DDR proteostasis, emphasizing both proteolytic and non-proteolytic mechanisms that modulate the stability, recruitment, and signaling dynamics of the DNA repair machinery. We discuss how disruption of this regulatory balance contributes to disease and outline therapeutic approaches targeting components of the ubiquitin system to restore balanced DDR signaling.

## 2. Ubiquitination Maintains Proteostasis of DDR Machinery

One important post-translational mechanism that regulates the stability, turnover, and availability of DDR factors is ubiquitination. Different forms of ubiquitin chains can be conjugated to DDR proteins to mediate either non-proteolytic signaling, which modulates protein activity, or proteolytic destruction via the proteasome. This dual regulatory mechanism ensures that certain DDR components are spatially retained and activated at DNA damage sites, while others are effectively eliminated once they are no longer required, preventing interference in DDR signaling [18].

Ubiquitin contains seven lysine (K) residues (K6, K11, K27, K29, K33, K48, and K63), which facilitate the assembly of structurally diverse mono- or polyubiquitination chains [9]. Modification of substrates with distinct ubiquitin chain linkages encodes different functional outcomes. Proteolytic ubiquitination regulates the lifetime and abundance of DDR proteins by targeting them for 26S proteasome-mediated degradation, predominantly via K48- or K11-linked ubiquitin chains assembled by specific E3 ubiquitin ligases. This process is crucial for the timely clearance of DDR regulators that might otherwise interfere with checkpoint control or DNA repair mechanisms [5,19]. In contrast, the non-proteolytic functions of the ubiquitin system are equally critical [9]. K63-linked ubiquitin chains frequently serve as a recruitment platform for downstream repair effectors at DNA damage sites and play important roles in protein quality control [9,20]. Recent studies have further expanded the concept of “ubiquitin code” in the DDR, identifying roles of K6 linkages in protein stabilization, K33 in trafficking regulation, and K27/K29 linkages as emerging modulators of DNA repair [20,21,22,23].

### 2.1. Proteolytic Regulation of DDR Machinery by Ubiquitination

The E3 ubiquitin ligase RNF138 exemplifies a typical case of proteolytic ubiquitination within the DDR. Upon recruitment of DNA double-strand break (DSB) sites, RNF138 promotes the ubiquitination of DNA repair proteins, including the Ku70/Ku80 complex. By assembling proteolytic ubiquitin chains, RNF138 triggers the proteasome-dependent removal of Ku proteins from DNA ends, a step that is essential for the proper progression of the repair pathway. This regulated breakdown prevents the persistent retention of Ku at damaged sites, which could otherwise sterically prevent subsequent repair processes. Thus, RNF138-mediated proteolysis functions as a critical “reset” mechanism for the DDR machinery [6,24].

Another well-characterized example of proteolytic ubiquitination in the DDR involves the E3 ubiquitin ligase MDM2. Under basal conditions, the tumor suppressor p53 is ubiquitinated by MDM2, maintaining low basal p53 levels via constitutive proteasomal destruction. Upon DNA damage, Inhibition of MDM2 activity leads to p53 accumulation, enabling the activation of apoptotic or cell-cycle arrest programs. This strictly regulated proteolytic axis guarantees that p53-dependent DDR signaling is selectively engaged in response to genotoxic stress [25,26]

SCF (Skp1–Cullin–F-box) E3 ubiquitin ligase complexes also play a key role in mediating proteolytic ubiquitination of DDR proteins. These complexes predominantly catalyze the formation of K48-linked ubiquitin chains that target substrates for proteasomal degradation, with substrate selectivity conferred by distinct F-box proteins. In the context of the DDR, SCF complexes promote the degradation of critical checkpoint regulators, such as CDC25A and Claspin, following DNA damage or during checkpoint recovery. Timely termination of checkpoint signaling via SCF-mediated degradation is essential to prevent sustained cell-cycle arrest and to enable the resumption of normal cellular functions [27,28].

### 2.2. Non-Proteolytic Regulation of DDR Machinery by Ubiquitination

Beyond degradation, the activation of DSB repair is critically dependent on non-proteolytic ubiquitination events [9,29]. RNF168 is a RING-type E3 ubiquitin ligase that plays a central role in amplifying and enhancing ubiquitin-dependent DDR signaling by catalyzing the conjugation of K27- and K63-linked ubiquitin chains onto chromatin-associated histone H2A and H2A.X in response to DSBs [9]. These epigenetic modifications serve as a molecular scaffold for the recruitment of key DDR modulators, including 53BP1 and BRCA1, to the DNA damage sites [5,29,30].

Although RNF168 is best known for ubiquitinating H2A-type histones during DSB repair, H1-type histone is also modified during this process through the coordinated action of the E3 ligase RNF8 and the E2 conjugating enzyme UBC13 [9]. RNF8 and UBC13 mediate K63-linked ubiquitination of H1 histones, creating an initial binding platform that triggers RNF168 recruitment to DNA damage sites following DSB induction. RNF168 recognizes these K63-linked ubiquitin chains via its ubiquitination-dependent DSB recruitment module 1 (UDM1) domain and subsequently catalyzes H2A ubiquitination at K15 at damaged sites, promoting the activation of downstream DSB response and repair pathways [31].

Similarly, the E3 ubiquitin ligase RAD18, together with the E2 enzyme RAD6, mediates monoubiquitination of proliferating cell nuclear antigen (PCNA) at stalled replication forks to facilitate translesion synthesis (TLS) without inducing proteasomal degradation after DNA damage. When replication forks stall, replication protein A (RPA) recognizes, and coats exposed single-stranded DNA (ssDNA) to stabilize the fork and prevent collapse [32]. RAD18 recognizes the RPA-coated ssDNA structure, binds to the RPA-ssDNA complex, and subsequently recruits RAD6. RAD18/RAD6 complex then works together to transfer a single ubiquitin to PCNA at K164 [33]. Monoubiquitinated PCNA serves as a binding site for TLS polymerases, recruiting high-fidelity replicative polymerase to resume DNA synthesis and allowing replication to proceed beyond DNA-damaged sites [34]. Activation of TLS is crucial to preventing prolonged replication fork stalling and collapse, therefore suppressing DSB formation and large-scale chromosomal instability [32,34,35]. Through this mechanism, RAD18/RAD6-dependent ubiquitination supports TLS as a DNA damage tolerance pathway that preserves genomic integrity [34].

### 2.3. Dysregulation of Ubiquitination in DDR and Its Relevance to Disease Progression

Dysregulation of the ubiquitination process is increasingly recognized as a key driver of defective DDR signaling and progressing disease. Ubiquitination regulates the stability, location, and activity of DDR proteins through both proteolytic and non-proteolytic mechanisms, facilitating precise detection, signaling, and repair of DNA lesions. Precise ubiquitin signaling at DSB sites is crucial for coordinating repair pathway choice and maintaining genomic integrity. Disruption of this regulatory layer compromises repair fidelity and promotes genomic instability, a defining hallmark of cancer [36].

**Figure 2:**
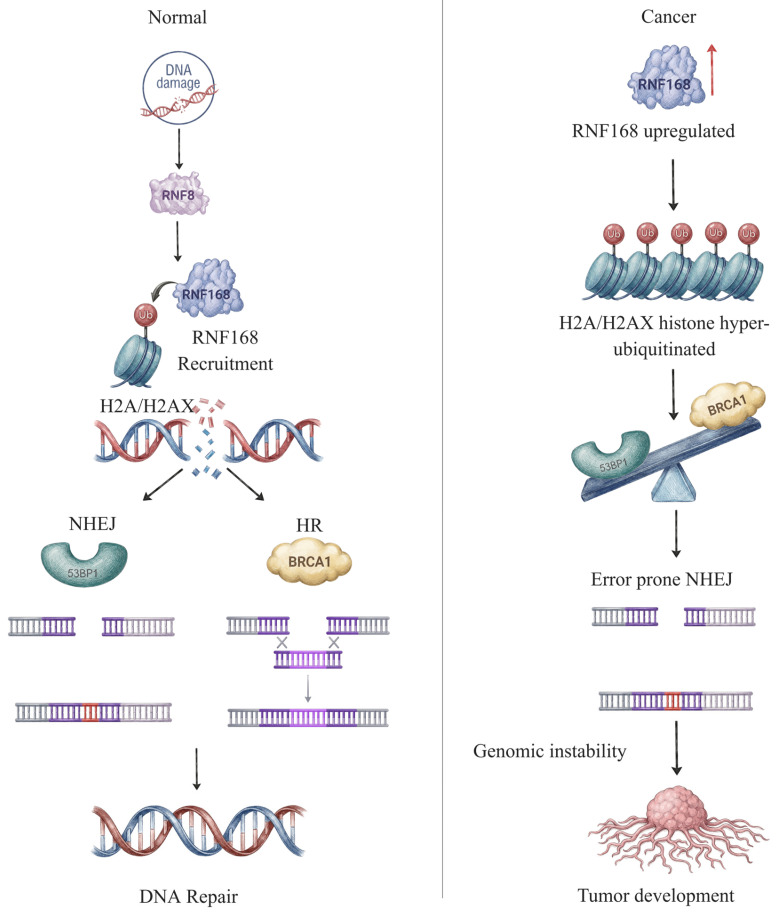
**RNF168 regulation in normal conditions and tumor development.** Under normal conditions, RNF168, a RING-type E3 ubiquitin ligase, acts downstream of RNF8 to mediate controlled mono- and K63-linked ubiquitination of histone H2A/H2AX. This ensures balanced recruitment of 53BP1 and BRCA1, supporting appropriate repair pathway choice between HR and NHEJ, thereby maintaining genome stability and DDR proteostasis. In cancer, RNF168 overexpression drives excessive histone ubiquitination and persistent 53BP1 accumulation suppresses BRCA1 recruitment and biases repair toward error-prone NHEJ, thereby promoting genomic instability and tumor development.

Dysregulation of RNF168, particularly through overexpression, disrupts the tightly maintained balance of ubiquitin-dependent DDR signaling (Figure 2). Excess RNF168 activity increases H2A/H2AX ubiquitination, resulting in aberrant and prolonged accumulation of 53BP1 and impaired recruitment of BRCA1 at DNA damage sites. This imbalance biases the repair pathway selection towards error-prone non-homologous end joining (NHEJ) while suppressing homologous recombination (HR), resulting in genomic instability. In cancer cells, particularly esophageal squamous cell carcinoma, RNF168 overexpression converts a normally protective DDR amplifier into a driver of mutagenesis repair and tumor development [30].

The E3 ubiquitin ligase NEDD4-1 modulates the proteolytic regulation of key tumor suppressors and signaling pathways, thereby contributing to dysregulated ubiquitination in the DDR. Under pathological conditions, NEDD4-1 activation promotes K48-linked ubiquitination and proteasomal degradation of PTEN, a key negative regulator of the PI3K/AKT pathway. Loss of PTEN stability results in persistent PI3K/AKT signaling, which enhances survival signaling, suppresses DNA damage checkpoints, and permits cell division despite unresolved genomic lesions. This proteolytic imbalance impairs DDR proteostasis by shortening the effective duration of checkpoint control and favoring proliferation over repair, which in turn promotes tumor development across multiple malignancies, including lung cancer [5,37].

Similarly, TRC8 (also known as RNF139), a RING-type E3 ubiquitin ligase, regulates the proteolytic turnover of proteins involved in growth and stress signaling, which contributes to ubiquitination control in the DDR. In addition to NEDD4-1-mediated PTEN degradation, TRC8 functions as a tumor suppressor by boosting ubiquitin-dependent degradation of substrates that cause uncontrolled proliferation. Loss or inactivation of TRC8, which has been reported in renal cell carcinoma, disrupts this proteolytic regulation, leading to stability of pro-proliferative signaling pathways. As a result, DDR checkpoint enforcement is compromised, allowing cells to divide even after DNA damage, which promotes genomic instability and tumor growth [37,38].

HERC2 is a critical E3 ubiquitin ligase in DDR signaling, and its dysregulation has been implicated in numerous malignancies. After DNA double-strand breaks, HERC2 is recruited to the γH2AX-MDC1 platform, where it stimulates RNF8 oligomerization and RNF168-dependent histone ubiquitination. This activity is essential for proper accumulation of DDR factors, including 53BP1 and BRCA1. Somatic mutation and amplification of HERC2 have been seen in uterine corpus endometrial cancer, skin cutaneous melanoma, lung adenocarcinoma, and sarcoma, leading to impaired DDR proteostasis and increased genomic instability. Furthermore, aberrant regulation of the MDM2-p53 axis by HERC2 enables damaged cells to evade checkpoint control, thereby promoting tumor initiation and progression [36,39].

## 3. Deubiquitination Maintains Proteostasis of DDR Machinery

To maintain proteostasis of the DDR machinery, ubiquitin signals must be precisely balanced. Deubiquitylation represents a critical regulatory process that fine-tunes ubiquitin signaling throughout the DDR. Deubiquitinating enzymes (DUBs) play an essential role in this process by counteracting ubiquitin-dependent proteasomal modifications of DDR proteins through both proteolytic and non-proteolytic mechanisms, preventing inappropriate proteasomal degradation and ensuring proper DDR signaling [40,41].

### 3.1. Proteolytic Regulation of DDR Machinery by Deubiquitination

One of the most well-characterized DUBs with this function is USP7. USP7, also known as HAUSP, is a ubiquitin-specific protease that prevents ubiquitin-dependent proteasomal degradation by removing K48-linked polyubiquitin chains from its substrates. One of its best-known targets is the p53 protein, which is stabilized and accumulates upon deubiquitination. The p53 stabilization is essential for its transcriptional program that promotes cell cycle arrest and DNA repair, playing a crucial role in determining cell fate. In addition to p53, USP7 also regulates the stability of Claspin by similarly preventing its degradation. Stabilized Claspin is essential for sustained checkpoint activation and efficient DNA repair [42].

Another deubiquitinating enzyme involved in this regulatory process is USP13, which controls the stability of TOPBP1, a central scaffold protein required for coordinating checkpoint activation, particularly in the ATR–CHK-mediated replication stress response. Given the pivotal role of TOPBP1 in sustaining DNA damage-induced cell-cycle checkpoints, its protein stability is critical for effective DDR signaling. USP13 removes degradative ubiquitin chains from TOPBP1, preventing its ubiquitin-dependent proteasomal degradation. By stabilizing TOPBP1, USP13 ensures proper checkpoint activation, preserves DDR fidelity, and safeguards genome stability [43,44].

Another example is USP11, a deubiquitinase that regulates both chromatin organization and the stability of key repair factors, including 53BP1. USP11 counteracts the ubiquitin-dependent proteasomal degradation of 53BP1, maintaining sufficient 53BP1 levels for its recruitment to DNA DSBs. Proper USP11 activity ensures controlled accumulation of 53BP1 at damage sites, promotes DNA end protection, and restricts excessive DNA end resection. Through this regulation, USP11 contributes to appropriate DNA repair pathway choice and supports genome stability during the DDR [45].

Also, within the USP family, USP28 is a cysteine protease deubiquitinase that regulates p53 stability in coordination with 53BP1. Upon DNA damage, USP28 is recruited to damage sites by the tandem BRCT domains of 53BP1, where it directly deubiquitinates p53 and prevents its ubiquitin-dependent proteasomal degradation. This stabilization of p53 preserves its protein levels, ensuring p53-dependent transcriptional activation of targets such as p21, maintaining G1 checkpoint fidelity, and thereby supporting genome stability during stress responses, including centrosome loss or ionizing radiation [46,47].

### 3.2. Non-Proteolytic Regulation of DDR Machinery by Deubiquitination

USP1 is a member of the USP family and is known for its role in deubiquitinating monoubiquitination signals. In the Fanconi anemia (FA) DNA repair pathway, USP1, together with its cofactor UAF1, removes the monoubiquitin signal from the FANCI-FANCD2 heterodimer on chromatin. This deubiquitination is required for dissociation of the FANCI-FANCD2 complex from DNA upon completion of repair, enabling proper restart of the replication machinery [48].

BRCC36, a component of the BRCA1-A complex, functions as a K63-linked deubiquitinase at DNA DSBs. By selectively removing ubiquitin from damaged chromatin, BRCC36 fine-tunes ubiquitin signaling to ensure stable anchoring of the BRCA1-A complex. This promotes controlled retention of the BRCA1-A complex at the damaged sites and limits excessive DNA end resection, thereby contributing to the maintenance of genome stability [49,50].

ATXN3 is a deubiquitinase that primarily regulates non-degradative ubiquitin signaling. It edits and trims K63-linked ubiquitin chains on chromatin, modulating the assembly and disassembly of DDR complexes. ATXN3 interacts with DDR scaffolding proteins such as MDC1, and checkpoint regulators including CHK1, contributing to precise ubiquitin signal refinement at damage sites. Through this non-proteolytic deubiquitination activity, ATXN3 coordinates the dynamic organization of DDR complexes, promotes timely checkpoint and repair signaling, and prevents prolonged or excessive DDR activation [51,52].

### 3.3. Dysregulation of Deubiquitination in DDR and Its Relevance to Disease Progression

Precise regulation of deubiquitination is critical for maintaining the proteostasis of DDR factors. Dysregulation of deubiquitinating enzymes can disrupt DDR signaling dynamics, leading to defective repair resolution, prolonged checkpoint activation, or aberrant apoptosis. Such perturbations contribute to the development of human disease, including cancer and metabolic disorders [41].

USP7 has been reported to be overexpressed in several types of cancer, including multiple myeloma, gliomas, neuroblastoma, and ovarian cancer, where its elevated activity promotes tumor cell survival. Mechanistically, USP7 stabilizes the E3 ubiquitin ligases MDM2 and MDMX through deubiquitylation, leading to suppression of p53. This activity impairs p53-dependent checkpoint control, thereby facilitating genomic instability [53,54,55].

In addition, USP7 is upregulated in cervical cancer, and its expression correlates with poor patient prognosis. Upon DNA damage, USP7 directly associates with the MRN–MDC1 complex and deubiquitinates MDC1, promoting its stabilization and sustained accumulation at sites of DNA DSBs. Stabilization of MDC1 facilitates efficient assembly and retention of the MRN–MDC1 signaling platform, resulting in prolonged activation of the DSB response pathway and enhanced survival and proliferation of cancer cells [56].

USP1 forms an active deubiquitinating complex with its cofactor UAF1 and can associate with RAD51AP1 to regulate key DDR substrates, including PCNA and the FANCI–FANCD2 heterodimer. The loss of USP1 activity directly impairs FANCD2 deubiquitination, resulting in its prolonged monoubiquitination. These modifications sustain DDR signaling, leading to defective repair resolution, enhanced replication stress sensitivity, chromosomal abnormalities, and cellular dysfunction, including bone marrow failure and hypersensitivity to DNA crosslink agents—hallmarks of the FA phenotype [57].

In contrast, USP1 dysregulation has also been observed in chronic stress conditions that induce persistent DNA damage. Sustained USP1 activity in this context prolongs DDR signaling. Persistent activation of DDR pathways favors apoptotic responses rather than successful repair, promoting β-cell apoptosis. Loss of β-cell mass consequently reduces insulin production and contributes to the development of diabetes [58,59].

**Figure 3:**
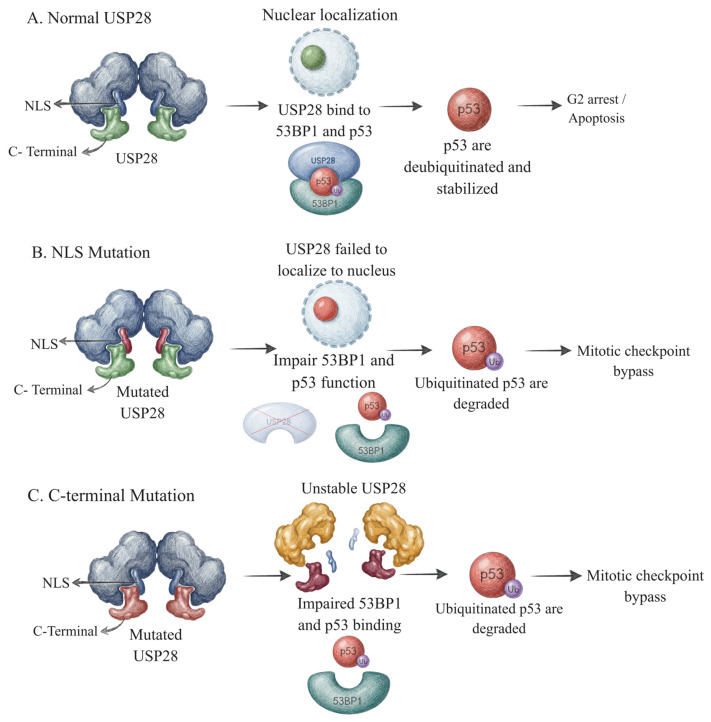
**Mutations in USP28 disrupt p53 stabilization and mitotic checkpoint control.** (**A**) Wild-type USP28, a deubiquitinase that is involved in DDR signaling, enters the nucleus via its NLS and forms a complex with 53BP1 to deubiquitinate and stabilize p53, thereby activating the mitotic surveillance checkpoint after prolonged mitosis. (**B**) NLS-deficient USP28 fails to localize to the nucleus, disrupting its interaction with 53BP1 and p53, resulting in p53 degradation and checkpoint bypass. (**C**) The C-terminal USP28 mutation destabilizes the protein, impairs nuclear accumulation, and prevents 53BP1 complex formation, resulting in loss of p53 stabilization and defective mitotic surveillance.

Dysregulation of the deubiquitinase ATXN3, particularly by polyglutamine expansion, impairs its DUB function and disrupts signaling required for proper regulation of the DDR, specifically in post-mitotic neurons. Consequently, ubiquitin signals persist at damage sites, leading to defective repair resolution and accumulation of unrepaired DNA lesions. In post-mitotic neurons, this persistent DNA damage triggers chronic DDR activation and cellular dysfunction, ultimately resulting in progressive neurodegeneration characteristic of spinocerebellar ataxia type 3 (SCA3) [60,61]. Dysregulation of USP28, particularly through cancer-associated mutations, disrupts critical structural features required for mitotic surveillance, including C-terminal interaction with 53BP1, USP28 dimerization, nuclear localization, and protein stability (Figure 3). Therefore, mutant USP28 fails to suppress p53 ubiquitination, leading to reduced p53 stabilization and impaired activation of p53-dependent growth arrest, which allows cells to continue proliferating despite mitotic errors. This defective mitotic surveillance permits the accumulation of chromosomal instability and mitotic defects, promoting tumor progression rather than triggering the elimination of genomically compromised cells. Importantly, loss of USP28 tumor-suppressive function enables cancer cells to evade mitotic checkpoints without broadly disrupting canonical DNA damage responses, highlighting a distinct mechanism by which USP28 dysregulation contributes to disease progression [62].

## 4. Clinical Implications

Targeting ubiquitin signaling represents a promising therapeutic strategy for a broad range of diseases, as ubiquitination and deubiquitination play essential roles in regulating DDR signaling. The therapeutic rationale of targeting the ubiquitin system in DDR is fundamentally based on the principle of synthetic lethality. Pharmacologically, inhibition of specific E3 ligases or DUBs can exploit the pre-existing genetic vulnerabilities of cancer cells, such as defects in HR. This approach creates a therapeutic window in which cancer cells, already compromised in one repair pathway, undergo catastrophic genomic instability upon disruption of a second, ubiquitin-regulated compensatory mechanism, while sparing normal cells with intact repair capacity.

Modulation of ubiquitin ligases and DUBs influences the ubiquitin-dependent recruitment and retention of DDR factors, alters repair pathway choice, and perturbs checkpoint signaling. Consequently, inhibition of selected ubiquitin ligases or DUBs can sensitize cancer cells to conventional treatments such as chemotherapy and radiotherapy, while potentially minimizing toxicity to normal tissues through tumor-specific synthetic lethal interactions. Table 1 provides a comprehensive summary of specific inhibitors and their corresponding target.

### 4.1. Therapeutic Drugs Targeting Ubiquitination in DDR

#### 4.1.1. E1 Inhibitor

TAK-243, also known as MLN7243, is a selective inhibitor of UBA1 that blocks the initiation of the ubiquitination cascade. TAK-243 inhibits UBA1, depleting cellular ubiquitin conjugates and disrupting ubiquitin-dependent signaling and DNA repair, leading to cell-cycle arrest and cancer cell death. It binds the UBA1 adenylation site to form a stable UBA1-ubiquitin adduct, blocking ubiquitin activation and transfer to E2 enzymes and triggering proteotoxicity and ER stress. Preclinical studies have demonstrated that TAK-243 exhibits potent antitumor activity in xenograft models and sensitizes cancer cells to genotoxic stress, highlighting E1 enzyme inhibition as a potential strategy to exploit ubiquitin-mediated DDR dependence in cancer [63,64]. However, preclinical work also shows that drug efflux transporters such as ABCB1 can limit TAK-243 cytotoxicity and may contribute to resistance in multidrug-resistant cancers, indicating potential challenges for effective therapy [84,85].

#### 4.1.2. E2 Inhibitor

NSC697923 is an inhibitor of the E2 ubiquitin-conjugating enzyme UBE2N (Ubc13), which catalyzes K63-linked ubiquitination. It covalently targets the active-site cysteine of UBE2N, blocking K63-linked ubiquitin chain formation needed for RNF8/RNF168-dependent DSB signaling. This prevents recruitment of repair factors like 53BP1 and BRCA1, weakening DSB repair and increasing replication stress. In addition, UBE2N mediates K63-linked polyubiquitination of PCNA, promoting error-free post-replication repair. Preclinical studies have shown that NSC697923, when combined with CX-5461, sensitizes HCT116 colon cancer cells to CX-5461 treatment [65]. As of current knowledge, NSC697923 remains a preclinical research inhibitor, with available evidence limited to cell-based studies. To date, no clinical development or human toxicity data have been reported for this compound [86].

#### 4.1.3. E3 Inhibitor

Among ubiquitin pathway-targeting agents, E3 ubiquitin ligase inhibitors are the most well-studied, particularly those targeting the MDM2 antagonist. Key molecule compounds include: Nutlin 3 and its derivative, Idasanutlin (RG7388), which are selective small-molecule inhibitors of MDM2, the E3 ubiquitin ligase responsible for ubiquitination and proteasomal degradation of the tumor suppressor p53. By disrupting the MDM2–p53 interaction, Idasanutlin prevents p53 ubiquitination and turnover, resulting in p53 stabilization and activation of downstream transcriptional programs that drive cell-cycle arrest and apoptosis.

Clinically, Phase I/II studies in patients with relapsed or refractory acute myeloid leukemia (AML) have demonstrated pharmacodynamic activation of the p53 pathway and measurable antileukemic activity following Idasanutlin treatment. Notably, higher baseline MDM2 expression in leukemic blasts correlates with improved clinical responses, reinforcing the therapeutic rationale of stabilizing p53 by preventing its ubiquitination and degradation, and providing direct clinical evidence that targeting E3 ligase–mediated ubiquitination is a feasible and biologically effective therapeutic strategy in cancer. However, clinical benefits are largely limited to tumors retaining wild-type p53, and dose-limiting gastrointestinal and hematologic toxicities have complicated optimization [67,87].

### 4.2. Therapeutic Drugs Targeting Deubiquitination in DDR

#### 4.2.1. USP1 Inhibitor

SP-002 (NCT06344052) has been reported as a potent and selective inhibitor of USP1, with potential application as either a monotherapy or in combination with the PARP1 inhibitor, olaparib. Inhibition of USP1 by SP-002 increases PCNA ubiquitination, thereby impairing DNA replication and inducing S/G2 cell-cycle arrest, ultimately suppressing tumor growth. Notably, the combination treatment of SP-002 and PARP inhibition synergistically induces cell death in homologous recombination-deficient (HRD) cell lines, leading to enhanced replication stress-induced apoptosis. This drug also exhibits low hematologic toxicity, highlighting USP1 inhibition as a promising therapeutic strategy, although clinical data are still limited [72].

In addition, Pimozide (NCT00004652) is an FDA-approved drug that is traditionally used for Tourette’s syndrome and schizophrenia [88,89]. Pimozide has been identified as a functional inhibitor of USP1 and has been investigated in multiple preclinical studies. Recent evidence indicates that Pimozide treatment inhibits USP1 activity, leading to increased ubiquitination of MAX/MYC, reduced expression of MYC target genes, and suppression of lymphoma cell growth [90,91]. Moreover, MYC-driven cancers exhibit elevated replication stress and heightened reliance on DDR pathways, supporting the therapeutic potential of USP1 inhibition in exploiting DDR-associated vulnerabilities in these malignancies, although no clinical trials have yet established safety or efficacy in oncology [92].

#### 4.2.2. USP10 and USP13 Inhibitor

Spautin-1 is a potent and selective small-molecule inhibitor of USP10 and USP13 and has been widely used as a preclinical tool compound. Inhibition of USP13 by Spautin-1 enhances the sensitivity of ovarian cancer cell lines to PARP inhibitors, indicating a functional interaction between USP13 activity and the HR repair pathway. Furthermore, Spautin-1 treatment alone has been reported to attenuate ATR–CHK1 signaling, resulting in replication stress-associated cell death. Preclinical studies suggest that Spautin-1 may be effective in cancers reliant on the HR repair pathway. However, no clinical trials have been conducted, and treatment-associated toxicities are not available [44,76].

#### 4.2.3. Broad-Spectrum USP Inhibitors

VLX1570 (NCT02372240) is one of the first small-molecule deubiquitinase inhibitors to enter clinical evaluation and represents the clinical analog of b-AP15. VLX1570 primarily targets USP14 and UCHL5, which disrupts proteosome-associated deubiquitination, leading to the accumulation of polyubiquitinated proteins and proteotoxic stress that triggers apoptosis in tumor cells. While it was evaluated in a phase I clinical trial in patients with multiple myeloma. However, the study was terminated due to severe dose-limiting toxicity [93].

Conversely, YM155 (sepantronium bromide) was initially identified as a Survivin inhibitor and has been extensively investigated for its anticancer activity across multiple malignancies. Preclinical studies have demonstrated that YM155 suppresses tumor growth in several cancers, including prostate cancer, melanoma, breast cancer, and B-cell lymphoma, either as a monotherapy or in combination with other agents, and it has advanced to phase I and II clinical trials [94,95]. Moreover, accumulating evidence suggests that YM155 can disrupt ATR/ATM-dependent signaling and repress the FA repair pathway, resulting in impaired DNA repair capacity and increased sensitivity to genotoxic stress [78,96,97]. While early clinical trials have shown that this drug is generally well-tolerated, detailed efficacy and toxicity profiles in patients have yet to be fully established.

## 5. Challenge and Perspective

Proteostasis is a fundamental cellular process that is essential for the proper coordination of DDR signaling. Disruption of proteostasis can lead to the accumulation of damaged or misfolded proteins, ultimately contributing to genome instability and the development of multiple disease hallmarks, including tumorigenesis. Therefore, understanding how proteostasis interfaces with DDR regulation is essential for identifying effective therapeutic targets.

Ubiquitination and deubiquitination operate in a dynamic and tightly regulated balance to ensure accurate DDR signaling. This coordinated turnover of ubiquitin marks enables precise temporal and spatial control of repair factor recruitment and signal amplification. Perturbation of this balance can compromise DNA repair efficiency, alter checkpoint activation, and reduce cell survival following genotoxic stress. As emphasized throughout this review, pharmacological targeting of ubiquitin-conjugating and deubiquitinating enzymes represents a promising therapeutic strategy to selectively disrupt DDR signaling and enhance the efficacy of DNA-damaging therapies.

Despite growing evidence supporting the involvement of ubiquitin-mediated proteostasis in DDR regulation, the precise molecular mechanisms connecting proteostasis to specific DDR pathways remain poorly comprehended and require further investigation. Although numerous inhibitors targeting ubiquitin-related enzymes have been developed, their clinical translation has been limited by poor specificity, off-target effects, and restricted progression beyond preclinical studies. Another major challenge is that, while some of these inhibitors have demonstrated efficacy in particular cell types, their therapeutic applicability and safety have not yet been fully validated and assessed in clinical trials. The functional redundancy and compensatory signaling within the ubiquitin system may attenuate the efficacy of single-agent inhibition, thereby limiting durable therapeutic responses.

Another major challenge is that, while some of these inhibitors have demonstrated efficacy in specific cell types, their therapeutic applicability and safety have not been fully validated in clinical trials. Because ubiquitin-mediated proteostasis is essential for genome maintenance in normal proliferating tissues, systemic inhibition of ubiquitin enzymes carries a substantial risk of on-target toxicity. These safety liabilities complicate dose optimization and have contributed to challenges in clinical development of ubiquitin-targeting therapies, particularly in combination with DNA-damaging agents.

However, recent studies provided encouraging evidence supporting ubiquitin-mediated proteostasis as a viable therapeutic target in DDR. Several inhibitors targeting ubiquitin pathways have demonstrated promising efficacy in preclinical and early-phase clinical trials. Additionally, combination strategies that integrate ubiquitin pathway inhibitors with DNA-damaging agents offer a synergistic approach, enhancing cancer cell sensitivity to genotoxic therapies or potentially overcoming therapy resistance. The successful translation of these findings into clinical practice will require the identification of reliable biomarkers capable of predicting patient response.

Looking forward, the paradigm of DDR-targeted therapy is shifting from traditional functional inhibition toward Targeted Protein Degradation (TPD), exemplified by Proteolysis Targeting Chimeras (PROTACs). In contrast to conventional small-molecule inhibitors, which rely on occupancy-driven pharmacology, PROTACs facilitate event-driven degradation of entire protein scaffolds. By utilizing heterobifunctional molecules, PROTACs confiscate endogenous E3 ligases, most notably Cereblon (CRBN) or Von Hippel–Lindau (VHL), which were previously described as key effector proteins, to promote the ubiquitination and subsequent proteasomal degradation of specific DDR targets. This approach is particularly advantageous for targeting DDR factors that lack enzymatic pockets or act primarily as structural scaffolds. For instance, DDR components can be selectively degraded by hijacking CRBN, thereby addressing the inherent limitations of catalytic inhibition and providing a robust strategy to counteract drug resistance in DNA-damaging therapies. By exploiting the endogenous ubiquitin-proteasome machinery, PROTACs provide a powerful complementary approach to conventional inhibitors for targeting the DDR and overcoming therapeutic resistance. However, PROTAC-based therapeutics often exhibit suboptimal pharmacokinetic properties, including poor oral bioavailability and limited cellular permeability, which represent major challenges for their clinical development.

Collectively, these findings highlight the central role of ubiquitin-mediated proteostasis in DDR regulation and its impact on genome stability, disease progression, and therapeutic intervention. While significant challenges remain in the development of selective and clinically effective inhibitors, continued efforts to elucidate the underlying mechanisms and optimize combination treatment strategies will be critical for advancing future therapeutic applications and improving patient outcomes across diverse malignancies.

## Figures and Tables

**Table 1:** Clinical and preclinical inhibitors targeting ubiquitin and deubiquitination pathways in the DDR.

Drug	Target	Clinical Status	Experimental Model	DDR Pathway Affected	Reported Toxicities/ Anticipated Safety Concerns
TAK-243 (NCT03816319)	UBA1	Phase 1 (recruiting)	Myelodysplastic Syndrome with Excess Blasts	Replication stress, Cell cycle checkpoints [63].	Preclinical tolerability: potential hematologic and hepatic toxicity anticipated (clinical DLTs under evaluation) [63,64]
NSC697923	UBE2N	Preclinical	Colon Cancer Models	Post replication repair, replication stress [65].	Toxic at sensitizing concentrations in normal cell (preclinical) [66]
Idasanutlin (RG7388; NCT02670044)	MDM2 (E3)	Phase 1/2	Acute Myeloid Leukemia	Cell cycle checkpoint regulation [67].	Gastrointestinal side effects (diarrhea, nausea, vomiting) [68]
Serdemetan (JNJ-26854165; NCT00676910)	MDM2	Phase I	Advanced Solid Tumors	Indirect modulation of DDR through p53 activation and S-phase arrest [69].	Dose-dependent QTc prolongation [69]
BI8622	HUWE1	Preclinical	Multiple Myeloma Cancer Models	HR [70].	Preclinical antiproliferative in vitro; no clinical data available [70,71]
SP-002 (NCT06344052)	USP1	Phase 1/2	Basal Cell Carcinoma	Replication stress [72].	Potential bone marrow affects assessed in vitro (CD34^+^ progenitor differentiation) [72]
RO7623066 (NCT05240898)	USP1	Phase 1	Advanced Solid Tumors	Replication stress/HR vulnerability [73].	Hematologic effects (anemia) [74]
XL309 (ISM3091; NCT05932862)	USP1	Phase 1 (recruiting)	Advanced Solid Tumors	Replication stress, HR [75].	No significant gastrointestinal or hematological toxicity (preclinical) [75]
Spautin-1	USP10/13	Preclinical	Ovarian Cancer Models	ATR-CHK1 signaling, PARPi sensitization [44,76].	No significant viability reduction in normal cells at ≤25 μM [77]
YM155	Broad-spectrum USP inhibitor	Preclinical	Breast Cancer Models	ATR/ATM signaling, FA repair pathway [78].	Hematologic suppression; fatigue; renal/hepatic/cardiac effects; electrolyte imbalance [79]
iRucaparib-AP6	PARP1 (PROTAC)	Preclinical	Breast/Ovarian Cancer	PARP1 degradation, DSB repair inhibition [80]	No dose-limiting toxicity observed in mild-to-moderate organ impairment [81]
ARV-825/dBET6	BRD4 (PROTAC)	Preclinical	Leukemia/Solid Tumors	Transcriptional control of DDR genes, NHEJ/HR [82]	No obvious systemic toxicity (preclinical) [83]

## Data Availability

No new data were created or analyzed in this study.
